# Elixir in Bronchial Irritation, Coryza and Catarrh

**Published:** 1904-12

**Authors:** 


### ELIXIR IN BRONCHIAL IRRITATION, CORYZA AND CATARRH.

| | | |
|---|---|---|
| ℞ | Atropine Sulphate | 1-10 of a milligramme. |
| | Terpine Hydrate | 1 gram. |
| | Benzo-Kinone | 4 grams. |
| | Heroine | 5 centigrams. |
| | Elixir de Garus (Codex) | 30 grams. |
| | Glycerine | 100 grams. |

M. F. Syrup.

Sig. One teaspoonful every two hours whenever the conditions are annoying, or three times a day between meals.

